# Pseudoaneurysm of the superior lateral genicular artery: case report of a rare complication after total knee arthroplasty

**DOI:** 10.1186/1754-9493-7-15

**Published:** 2013-05-20

**Authors:** Pramod Saini, Sanjay Meena, Rajesh Malhotra, Shivanand Gamanagatti, Vijay Kumar, Vaibhav Jain

**Affiliations:** 1 Department of Orthopedics, All India Institute of Medical Sciences, Ansari, Nagar, New Delhi 110029, India; 2 Department of Radiodiagnosis, All India Institute of Medical Sciences, Ansari, Nagar, New Delhi 110029, India

**Keywords:** Total knee arthroplasty, Stiff knee, Pseudo aneurysm, Genicular artery, Embolization

## Abstract

**Background:**

Pseudoaneurysm of superior lateral genicular artery following total knee arthroplasty is a rare complication and has been reported following lateral release performed for eversion of patella in a knee with tight lateral structures.

**Case presentation:**

This report describes a case of pseudo aneurysm of superior lateral geniculate artery that developed after primary Total knee arthroplasty for a stiff knee in a 68 year old patient. Patient presented with pain and rapidly increasing swelling in early post operative period. Diagnosis was made on duplex ultrasound and confirmed by angiography. Angiographic coil embolisation of the pseudoaneurysm was performed. Since no lateral release was performed in this case, the probable mechanism was shear injury to the vessel.

**Conclusion:**

Pseudoaneurysm of superior lateral genicular artery can occur in absence of lateral release by shear injury to an atherosclerotic vessel. Angiographic coil embolisation appears to be the best method for treating such post arthroplasty pseudoaneurysm because of less chance of infection, non interference with rehabilitation and diagnosis and treatment during same procedure.

## Background

Vascular complications after Total knee arthroplasty (TKA) are quite rare, thier incidence being 0.03% to 0.2% [1]. Pseudoaneurysms following TKA involving poplitieal artery, anterior tibial artery and geniculate arteries have been reported previously [2-9]. To the best of our knowledge no case of pseudoaneurysm following Total knee arthroplasty in a stxciff knee has been reported. Though rare, they can adversely affect the final outcome and may even be limb threatening. High index of suspicion may help in early identification and treatment of this potentially devastating complication. We report a case of 68 year old male who presented with rapidly increasing swelling in early post operative period following TKA for a stiff knee with 30° of flexion contracture. We present this case to discuss probable aetiology, diagnosis and management of this rare complication.

## Case presentation

**Figure 1:**
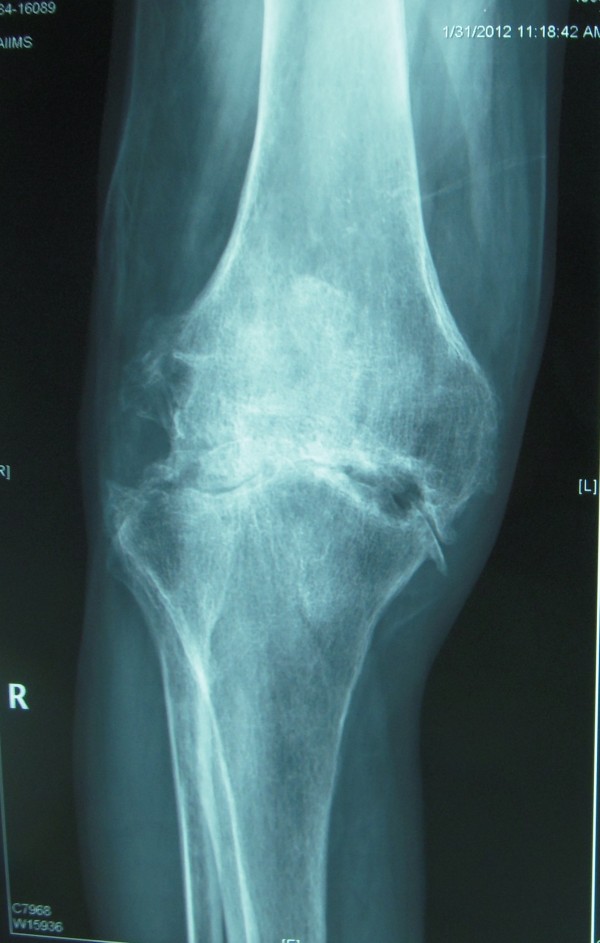
Preoperative AP radiograph of the right knee joint showing severe arthritis of the joint.

**Figure 2:**
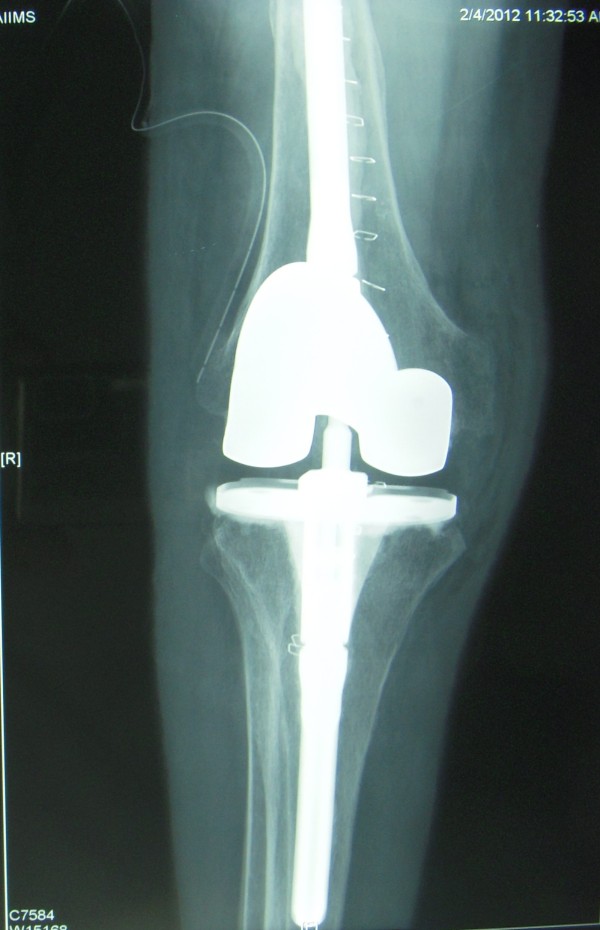
Postoperative AP radiograph of the right knee joint.

**Figure 3:**
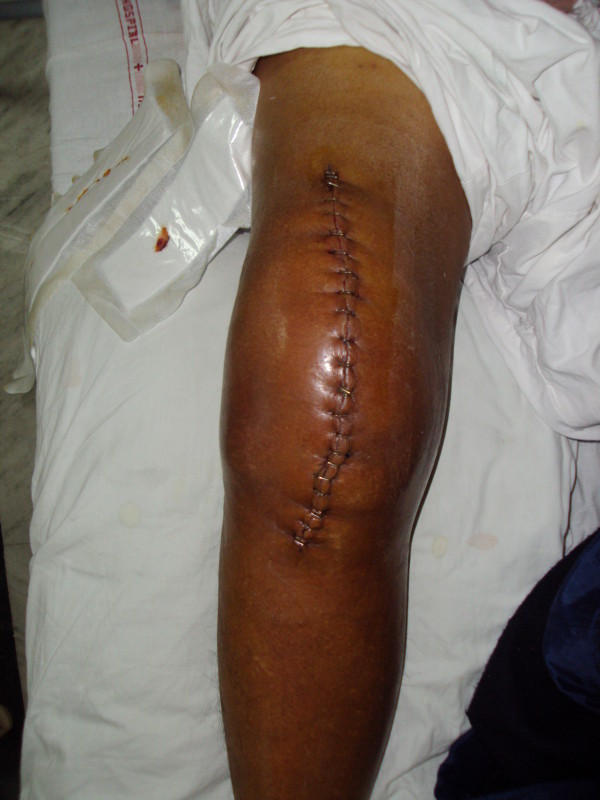
Clinical image of the patient’s knee showing massive swelling.

**Figure 4:**
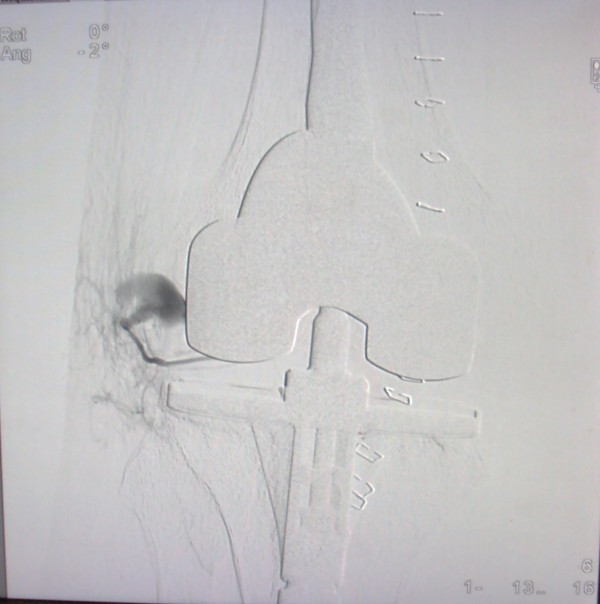
Intraoperative image from C arm showing pseudo aneurysm originating from superior lateral geniculate artery.

**Figure 5:**
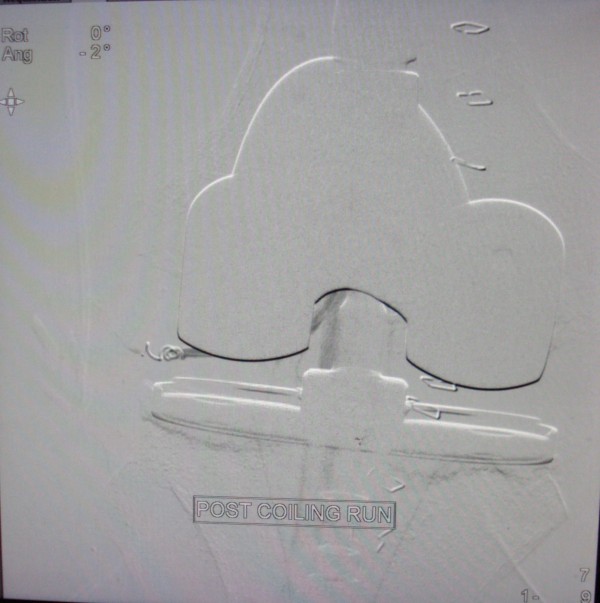
Image showing embolized superior lateral genicular artery.

A 68 year old man with stiff knee due to long standing osteoarthritis of underwent primary TKA (LCCK, Legacy Constrained Condylar Knee Nexgen, Zimmer, Warsaw,USA) (Figures 1 and 2). Preoperatively, he had flexion contracture of 30 degrees with only 10° of further flexion possible. There was a varus deformity of 10 degrees with no noticeable instability. Apart from long standing hypertension of 15 years, there was no other co morbidity. Due to stiffness of the joint, extensive soft tissue releases including excision of all fibrotic bands over patellofemoral and suprapatellar areas, debridement of medial and lateral gutters of the knee joints, excision of the lateral aspect of the prepatellar fat pad and quadriceps snip were performed to facilitate eversion of patella and exposure of the joint. Lateral retinaculum was left intact. There were no intraoperative complications and the alignment and stability of the prosthesis was satisfactory. Tourniquet was inflated before cementing for a total duration of 20 minutes. Before closure, tourniquet was deflated and hemostasis was achieved. Immediate postoperative period was uneventful and one unit blood was transfused to compensate for intra operative losses. Drain was removed 24 hrs after surgery. Static quadriceps exercises were started followed by knee mobilization on third day. On fourth post operative day, patient complained of increasing pain and swelling over the knee joint. The swelling was warm, tender, non pulsatile and any passive or active motion aggravated the pain. There was no neural deficit with good posterior tibial and dorsalis pedis pulses and normal capillary refill. Blood chemistry and coagulation profile were normal. Initially the limb was elevated and cold compresses were applied. Within the next two hours, swelling rapidly increased in size extending into distal thigh (Figure 3). Urgent Doppler ultrasound was performed which revealed pseudo aneurysm of size 3.5 × 2.3 cm^2^ arising from a vessel on lateral aspect of knee joint but its vessel of origin could not be identified. A subsequent angiogram revealed pseudo aneurysm originating from superior lateral geniculate artery (Figure 4). Using a micro catheter, a sub selective catheterization of the superior lateral genicular artery was performed and the artery was embolized by means of 3 mm coils (Figure 5). The patient experienced immediate relief from pain and swelling gradually decreased in size. The limb was immobilized for next 24 hours after which range of motion exercises were started as tolerated by the patient. Repeat Doppler 24 hr later showed no aneurysm with normal arterial and venous refill. There was a marked improvement in functional capabilities with range of motion of 5-100° and no evidence of recurrent bleeding at follow up.

## Discussion

A knee with preoperative arc of motion less than 50 deg° is considered to be stiff [10,11]. Eversion of patella is the most difficult step during TKA in such knees due to contracture and fibrosis of surrounding tissue needing additional steps like lateral release, quadriceps snip, quadricepsplasty, tibial tubercle osteotomy and also skeletaonization of the proximal medial tibia [10,11]. During these steps the surrounding neurovascular structures are at risk and can be damaged directly by instruments or indirectly by stretch and shear force.

Pseudoaneurysms following arthroplasty are rare with one study reporting incidence of 0.03% [12]. In contrast to true aneurysm, a pseudoaneurysm has a fibrous wall which under the effect of systemic pressure increases in size. It may compress surrounding structures causing distal neurovascular deficit or may rupture into surrounding tissues producing swelling [7], bleeding from suture line [8], calf pain and ecchymosis mimicking DVT [4] or acute and recurrent haemarthrosis [8,9]. Many mechanisms have been described such as-perforation by a retractor, injury by tourniquet, direct trauma to the vessel or secondary to the heat of the cement, repeated local trauma and injury to an atherosclerotic artery [3-7].

Pseudo aneurysms of superior lateral geniculate artery following TKA have been previously reported [5,7,8]. In these reports, lateral release performed during exposure was reported to be the cause [7,8]. The superior genicular arteries branch from the popliteal artery, curving round proximal to both femoral condyles to reach the anterior aspect of the knee. This artery is found within a triangle limited anteriorly by the vastus lateralis muscle, posteriorly by the short head of the biceps femoris muscle, and inferiorly by the lateral condyle of the femur. The lateral superior genicular artery passes under the tendon of biceps femoris and divides into superficial and deep branches. The superficial branch supplies vastus lateralis and anastomoses with the descending branch of the lateral circumflex femoral and lateral inferior genicular arteries, while the deep branch anastomoses with the medial superior genicular artery, forming an anterior arch across the femur with the descending genicular artery. The superficial branch is vulnerable if the lateral patellar retinaculum is divided surgically [13].

The probable mechanism in this case might be shear injury to the vessel resulting in intimal injury during manipulation or surgical exposure since lateral release was not performed. This assumption is strengthened by a recent report of ruptured pseudo aneurysm following manipulation under anaesthesia of a stiff total knee arthroplasty [14]. Since the patient had a long history of hypertension, the vessels may have been atherosclerotic making them prone to injury [3,4,6,7].

Aneurysm smaller than 2 cm in size can be managed with direct compression to induce thrombosis [6]. For those upto 5 cm in size, options are excision of aneurysm and ligation of feeding vessel [4,8], percutaneous thrombin injection [5] and intravascular coils and beads [3-9]. Surgical wound exploration requires additional anaesthesia and in the presence of an implant, it increases the risk of infection. Also, repeat surgery interferes with rehabilitation protocol. Ultrasound guided percutaneous thrombin injection and angiographic coil embolisation are minimal invasive alternatives. However, thrombin injections are associated with failures, propagation of thrombus and chances of infection through injection site. Angiographic embolisation appears to be the best method for these small vessels as it can be performed by percutaneous arterial catheterization of femoral artery under local anesthesia without any associated risk of infection. There is no need for altering rehabilitation programme and patient can be mobilised as soon as pain subsides. Most importantly, angiography provides the ability to diagnose and treat simultaneously.

## Conclusion

Though rare, soft tissue handling during primary total knee arthroplasty in a stiff knee can cause pseudo aneurysm. High index of suspicion should be maintained for this complication in post operative patients with acute swelling. Doppler ultra sound is the initial diagnostic method of choice with angiography being the definitive one. Intravascular coils provide a useful modality of treatment without any associated risk of infection or need of modifying rehabilitation protocol.

## Consent

Written informed consent was obtained from the patient for publication of this case report and accompanying images. A copy of the written consent is available for review by the editor-in-chief of this journal.

## Competing interests

The authors declare that they no competing interests.

## Authors’ contributions

RM conceived the idea. PS and SM wrote the manuscript. RM, VK, VJ operated the patient. SG performed coiling of the vessels. All authors contributed to and added to the manuscript. All authors read and approved the final manuscript.
